# Premotor biomarkers for Parkinson's disease - a promising direction of research

**DOI:** 10.1186/2047-9158-1-11

**Published:** 2012-05-31

**Authors:** Brian R Haas, Tessandra H Stewart, Jing Zhang

**Affiliations:** 1 Department of Pathology, University of Washington School of Medicine, HMC Box 359635, 325 9th Avenue, Seattle, WA, 98104, USA

**Keywords:** Parkinson’s disease, Biochemical markers, *LRRK2*, *GBA*, α-synuclein, DJ-1, Clinical biomarkers, Premotor, Neuroimaging, *parkin*, CSF

## Abstract

The second most serious neurodegenerative disease is Parkinson’s disease (PD). Over the past several decades, a strong body of evidence suggests that PD can begin years before the hallmark clinical motor symptoms appear. Biomarkers for PD are urgently needed to differentiate between neurodegenerative disorders, screen novel therapeutics, and predict eventual clinical PD before the onset of symptoms. Some clinical evaluations and neuroimaging techniques have been developed in the last several years with some success in this area. Moreover, other strategies have been utilized to identify biochemical and genetic markers associated with PD leading to the examination of PD progression and pathogenesis in cerebrospinal fluid, blood, or saliva. Finally, interesting results are surfacing from preliminary studies using known PD-associated genetic mutations to assess potential premotor PD biomarkers. The current review highlights recent advances and underscores areas of potential advancement.

## Introduction

**Figure 1:**
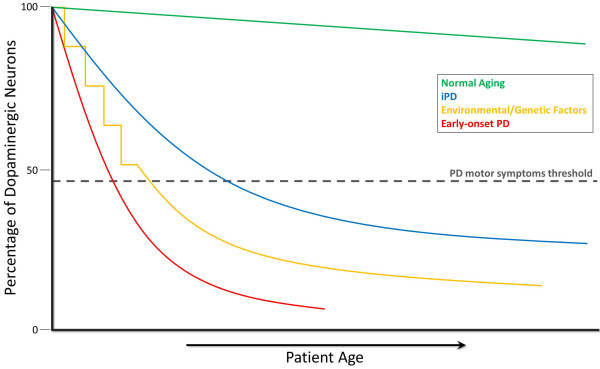
**Evolution of PD.** During the course of normal aging (green line), small but slow dopaminergic degeneration occurs without any motor symptoms. Typically idiopathic PD (iPD) (blue line) is of unknown origin but is thought to develop gradually over time with a slow degeneration of dopaminergic neurons leading to the emergence of the classic PD motor symptoms later in life. Another model of dopamingeric neurodegeneration leading to PD motor symptoms involves repeated exposure to environmental toxicants over time in combination with a genetic predisposition to dopaminergic neuron loss (yellow line). Early-onset PD (red line), as caused by mutations in the *parkin* gene, involves a precipitous decline in dopaminergic neuron number where PD motor symptoms can present decades prior to those in iPD. One more scenario (not shown) of PD-motor symptom development involves possible *in utero* environmental toxicants or genetic factors leading to an atypically low number of dopaminergic neurons at birth and increased susceptibility to PD development.

Parkinson’s Disease (PD) is a common and incapacitating neurodegenerative disease which affects the human population worldwide [1-3]. It generally presents clinically in adults over 60 years old, and major symptoms include akinesia, tremor, bradykinesia, and postural instability. Currently, its diagnosis relies largely on assessment of clinical symptoms. Unfortunately, this purely clinical diagnosis, combined with the similarities of PD symptoms with those of other neurodegenerative and movement disorders, results in frequent misdiagnosis: up to 50% of the time in primary care settings, while movement specialists fare somewhat better with an early PD misdiagnosis rate around 10% [4-6]. PD can currently only be confirmed through its pathological hallmark of Lewy bodies and Lewy neurites located in residual neurons or axons, respecitively, upon postmortem analysis [7]. To complicate matters, diagnosis is generally not made until an advanced stage of neurodegeneration: by some estimates, 50-60% of dopaminergic neurons in the substatia nigra are lost prior to the time of clinical diagnosis [8-13], limiting effectiveness of potential neuroprotective therapies due to the low number of remaining neurons (Figure 1).

Thus, introduction of new treatments and interventions for PD will depend on improved diagnosis, preferably at early, premotor stages at which a larger population of dopaminergic cells remains. Furthermore, the progression of the disease must be accurately assessed, in order to monitor efficacy of treatments and improve individualized therapeutic strategies. Much work toward this end has focused on identifying sensitive, specific biomarkers for PD. According to the NIH biomarker working group, a biomarker is “a characteristic objectively measured and evaluated as an indicator of normal biological processes, pathogenic processes or pharmacological responses to a therapeutic intervention”[14,15]. In other words, an ideal biomarker for PD must be able to identify almost all cases of PD in its premotor stages, change concomitantly with disease stages, and differentiate between PD and other neurodegenerative diseases. In practice, however, a combination of several biomarkers will likely be needed to correctly diagnose and track PD progression, and this review focuses on some of the most promising premotor PD biomarkers which could be most effective.

### Potential premotor PD biomarker candidates

#### Clinical biomarkers

It has been recognized recently that a variety of nonmotor symptoms associated with PD may manifest years before the onset of motor symptoms. Indeed, these premotor symptoms have been exploited in the last few years as possible premotor biomarkers for PD. Though not an exhaustive list, we focus here on some of the most promising potential clinical premotor PD biomarkers, namely, rapid eye movement (REM) behavior disorder (RBD), bowel dysfunction, olfactory deficits, and mood disorders.

It has been long observed that PD patients can have a variety of sleep disturbances, most frequently manifesting as RBD, which can precede the clinical motor symptoms associated with PD by several years [4,16-20]. In fact, recent findings indicate that RBD is one of the symptoms most strongly correlated with the presentation of synucleinopathy (including multiple system atrophy (MSA), PD, and dementia with Lewy bodies) later in life [19,21].

Disruptions in bowel movements are another known co-morbidity to PD. Much like RBD, constipation can arise many years prior to the motor symptoms of PD [22] and affects most PD patients [23]. Indeed, the frequency of bowel movements was correlated inversely with PD risk, and constipation may be one of the first symptoms of PD [22]. These findings were supported by an additional epidemiological study involving women in Olsmsted County, Minnesota, where an association between earlier life constipation and PD risk was well documented [24]. Together, these reports document the association of PD diagnosis with constipation symptoms emerging as much as 20 years earlier.

Olfactory disruption is also a common symptom associated with PD. Difficulties in detecting, discriminating, and identifying odors are observed in up to 90% of PD patients [25-27]. However, cross-sectional and longitudinal studies of PD patients have frequently found olfactory deficits to be largely uncorrelated with disease stage or severity [28-31], leading to the hypothesis that olfactory decline may occur largely prior to the onset of nonmotor symptoms. Indeed, as with constipation and sleep disturbances, olfactory deficits can occur in the several years leading up to motor impairments and clinical PD diagnosis [25]. This idea is further supported by the finding that, in recently diagnosed PD patients with mild symptoms, severity of olfactory deficits has been found to correlate with dopaminergic dysfunction as measured by SPECT, despite a lack of correlation between either measurement and severity of motor dysfunction [32-34]. To determine whether these early olfactory symptoms might prove useful as a premotor biomarker for PD, a number of studies have examined olfaction in early or asymptomatic patients. A recent prospective study has shown that patients with poor olfaction were more likely to develop PD in the 4-year follow up period than those with normal olfaction [35]. Additionally, another study found that when assessing asymptomatic first-degree relatives of PD patients, performance levels of odor discrimination robustly correlated with future PD risk [36]. Couple these studies with an initial report revealing that olfactory identification dysfunction on the University of Pennsylvania Smell Identification Test was able to distinguish PD from other movement disorders [26], and a strong candidate for a clinical premotor PD biomarker emerges.

Another potential clinical premotor PD biomarker is the presence of mood alterations. Some of the most common psychological symptoms associated with PD are depression, anxiety, and apathy [37]. Depression specifically is thought to be biological component of the disease (as opposed to a psychological reaction to the disease) as it is detected in nearly a quarter of patients in the early stages of PD [38,39]. Several groups have indicated that in the later stages of PD, depression rates are approximately 40%, but that this rate is probably grossly underestimated [40,41]. Further, examination of historical medical records at least 20 years before PD diagnosis led to the discovery that anxiety was associated with the later incidence of PD [42]. A few different groups have also speculated about a possible parkinsonian personality that involved such traits as inflexibility, neuroticism, obsessive-compulsivity, uneasiness, and anxiety, among others [43-47]. These characteristics are often present from childhood, leading to the possibility of long lasting biological changes preceding PD motor symptoms, and possibly implying that the observed motor symptoms of PD are really characteristic of end-stage disease. Supporting the idea that these symptoms are a component of the disease are findings of alterations in the serotonergic signaling pathways, long known to interact with the classic dopaminergic neuronal degeneration associated with PD and major target of anti-depressant medications. For example, a major serotonergic nucleus, the dorsal raphe, has enhanced cell loss in depressed PD patients compared to non-depressed PD patients [48]. Moreover, recent studies have shown a reduction of gray matter densities in the orbitofrontal cortex which are known to be involved in serotonergic pathways responsible for mood disorders [49,50]. Lewy body-related lesions also have been found in raphe nucleus of early stage PD patients and in asymptomatic individuals [51,52], further implicating a serotonergic pathology in PD depression. However, as with RBD, constipation, and anosmia, anxiety and depression are very nonspecific, and do not stand alone as clinical premotor PD biomarkers.

In short, a number of clinical symptoms appear years before the motor symptoms of PD. However, while each of the biomarkers occurs during premotor stages of PD, can be measured, and tracked, none is specific enough to be used alone as a premotor PD biomarker. As research progresses, the most likely role of these clinical premotor biomarkers will be a supportive one, serving to confirm the sensitivity and specificity of other premotor PD biomarkers. Future work should also focus on using a combination of clinical premotor biomarkers to enrich patient populations for neuroimaging and biochemical screening and assess in tandem multiple clinical symptoms for correlation for developing PD later in life, along with coupling premotor clinical signs with biochemical markers, neuroimaging, and genetic testing (see below for more discussion on the topic).

#### Biochemical markers

Examination of various tissues and body fluids for the relative levels of proteins and other molecules provides information about the state of the local system, including its metabolic state, disease condition, and response to therapeutic treatments. Therefore, measurement of a number of known small proteins and other molecules along with unbiased profiling (recently reviewed elsewhere [53,54]) is currently one of the most promising avenues for potential premotor biomarkers of PD (Table 1).

**Table 1:** Summary of possible premotor PD biomarkers

	Existing Biomakers			Potential Biomakers			Candidate biomakers	
	Uric acid	*a*-sysnuclein	DJ-1	SPECT	fMRI	PET	Aβ42	tau
Blood	74–76	63	82					
CSF		53, 54, 56, 59	53,79				130	130
Saliva		69,71						
Skin		67						
Glucose Binding						92		
Iron					100			
Structural Brain Elements					97–99			
DAT Binding				88–90, 93, 94				

##### a. α-Synuclein

α-synuclein has long been known to form protein aggregates found in Lewy bodies, and has been a major area of inquiry for PD treatments and biomarker discovery. Indeed, a consensus result of measuring the different forms of the protein that accumulate in cerebrospinal fluid (CSF), a body fluid in close proximity and interaction with the central nervous system (CNS), has started to emerge. Using both ELISA and Luminex assays, several groups have examined α-synuclein in CSF samples. These studies, which included large cohorts of patients, have shown a reduction of α-synuclein levels in CSF of PD patients offering a reasonable differential sensitivity and specificity compared to healthy controls [55,56]. However, there was no correlation between disease severity/duration and α-synuclein levels in these investigations, in contrast to a previous report which also detected reduced α-synuclein in the CSF of PD patients [57]. Notably, in smaller scale investigations, while several groups have also confirmed these observations [56,58,59], others reported no discernible differences in α-synuclein levels between control and PD patients [60,61]. Many issues are potentially responsible for these discrepancies, including sampling protocols, control for blood contamination (see below) and differences in detection/capture antibodies [62].

Because collection of CSF is somewhat invasive, multiple groups have investigated α-synuclein levels in other tissues as PD biomarkers. Due to its ready accessibility, blood has been the subject of a number of studies, but the results have been largely contradictory and inconclusive [55,63-66]. A recent bright spot appears to be the role of the concentrations of oligomeric α-synuclein in plasma samples. El-Agnaf *et al.* discovered that the concentration of oligomeric forms was increased in PD patients compared to controls [65]. They achieved high specificity (85%) for the differentiation of PD patients versus controls despite significant overlap of concentrations with the control group [65]. Another group has recently demonstrated significantly higher levels of auto-antibodies towards monomeric α-synuclein in the blood sera of PD patients compared to those from the control group [67]. However, measurement of α-synuclein in blood remains a significant problem, since >98% blood based α-synuclein is contained in red blood cells, making hemolysis, *in vitro* or *in vivo*, a major confounder in these studies [55,68]. Therefore, potential biomarkers based on blood α-synuclein remain either disappointing or too preliminary to be clinically useful.

Several investigators are pursuing α-synuclein levels in other tissue types as potential premotor PD biomarkers. For example, one initial report has detected α-synuclein in skin fibroblasts [69]. This group demonstrated that α-synuclein levels are elevated in the skin fibroblasts of PD patients compared to controls [69]. Although this study involved a small number of subjects and has yet to be replicated, using skin as a premotor biomarker has potentially high value and utility as it is highly accessible and can be procured without more invasive procedures.

Another promising line of evidence has emerged for using α-synuclein as a premotor PD biomarker in human saliva. Abnormal saliva production has been a long shown to correlate with PD severity and known to be a prevalent nonmotor symptom [70]. Del Tredici *et al*. recently examined post-mortem PD, MSA, and control submandibular glands for the presence of aggregated α-synuclein [71]. They discovered the presence of Lewy Body pathology by immunohistochemistry in the submandibular glands of all the PD cases examined, but not the control or MSA cases [71] . Because the submandibular glands respond to parasympathetic innervations from the medulla [72], the cells responsible for saliva secretion possess direct contact with the CNS. Therefore, any saliva secreted from these glands may provide indirect access to α-synuclein levels of the CNS. Saliva, as a biological fluid, is completely accessible, can be obtained without any invasive procedures, is readily available in large quantities, and can be sampled virtually an unlimited number of times from any individual. For these reasons, we are currently investigating α-synuclein in saliva as a biomarker. We are able to detect α-synuclein levels in human saliva [73] and are investigating whether levels of salivary α-synuclein differ between PD and healthy controls. Clearly, this promising line of research will require independent validation from several groups before its adaptation as a clinically useful biomarker.

##### b. Anti-oxidative stress makers

Besides α-synuclein, another major biochemical pathway dysregulated in PD is the elevation of oxidative stress [74]. Accordingly, several groups have explored oxidative stress metabolites, such as uric acid [75], and proteins as PD biomarkers. Three independent, large studies have indicated that higher levels of uric acid in serum confer lower risk of developing PD [76-78]. Additionally, the subset of diagnosed PD patients with higher uric acid levels progressed more slowly [79]. Higher uric acid levels however, though correlated with decreased risk [79], do not provide enough information to be pursued further as a premotor PD biomarker. This is mostly due to the largely non-specific action of uric acid as an anti-oxidant. Uric acid levels are tightly controlled by the body and, most importantly, while decreased risk may correlate with higher levels of uric acid, PD can still develop despite higher uric acid levels.

Similarly, the protein DJ-1 is expressed at high concentrations in cells and is thought to act as an antioxidant [80,81] and interacts with many of the same proteins as α-synuclein [82]. An initial study showed that DJ-1 is secreted into the plasma of both normal and PD patients [83]. However, no significant differences between PD and control groups were observed, nor was any correlation with age. This initial study set the stage for future studies examining the levels of DJ-1 in CSF and blood plasma. Waragai *et al*. demonstrated several years ago that DJ-1 levels are increased in the plasma of PD patients [84]. The same group also showed an increase of DJ-1 in the CSF of PD patients compared to control patients [85]. However, a more recent study has examined the levels of DJ-1 in PD patient CSF and has come to the opposite conclusion. It found that DJ-1 levels actually significantly decreased in PD patient CSF using a highly sensitive Luminex assay while at the same time controlling for possible blood contamination of CSF, which can confound interpretation and reliability of results [55]. The same group of investigators also concluded that in the same cohort of patients and controls, while CSF DJ-1 levels decreased, plasma DJ-1 was largely unaltered [55]. Much like α-synuclein, the discrepancy among different investigations is attributable to many issues, particularly hemolysis [68]. As oxidized isoforms of DJ-1 are indicative of increased oxidative stress [86-88], moving forward, they should be explored along with total DJ-1 and other promising biomarkers, in various body fluids, especially in the saliva of PD patients [73] or those with premotor signs, thereby facilitating the development of premotor biomarkers.

#### Neuroimaging

As technology has advanced in the past several decades, it has allowed clinicians and researchers to acquire more reliable and higher resolution data. Several techniques are being investigated for their use as potential premotor biomarkers [89]. Here, we will examine single-photon emission computed tomography (SPECT), positron emission tomography (PET), and functional magnetic resonance imaging (fMRI) (Table 1).

SPECT has been used for some time to image the dopamine transporter (DAT). It has routinely established a reduced binding in patients with PD [90] and can aid clinicians in diagnosis and monitoring, as the decreased ligand binding to the DAT correlates with disease progression and clinical scoring [91,92]. Although long term pre-symptomatic screening with DAT SPECT for PD is not particularly feasible due to high costs, limited access, and general difficulty of working with radioactive tracers, one group has shown it is useful for distinguishing between PD and other parkinsonian-like conditions [93]. Additionally, some PET imaging has shown reduced binding of glucose in posterior parietal and cortical gray matter of PD patients with dementia [94]. PET can also be used as a highly sensitive measure to assess dopamine uptake and binding to the DAT. Accordingly, PET imaging, which assesses radiolabeled ligands for DAT, can be utilized to distinguish premotor PD [95,96]. Additionally, fMRI is another potential neuroimaging modality for use as a premotor biomarker for PD. It has been known almost a decade that whole brain atrophy is precipitated in PD versus controls and slightly less than in Alzheimer’s disease [97]. More recent work has demonstrated that irregularities in the substantia nigra of early PD patients correlate with an increase in iron, which is a known to be related to neuronal loss [98]. In addition, one group has been able to provide 100% sensitivity and specificity for differentiating PD patients from healthy controls when imaging the caudal substantia nigra [99]. Another recent work has arrived at a 95% accuracy in distinguishing PD from healthy controls using multimodal MRI of subcortical gray matter [100]. While fMRI can successfully separate PD patients from controls, further volumetric fMRI characterization of PD and related disorders is currently underway and needed to provide insight into whether fMRI can serve as an effective premotor biomarker [101].

The neuroimaging markers reviewed all are able distinguish diseased PD patients from normal controls and have the ability in some situations to identify probable PD in premotor stages. However, their usefulness is limited due to issues of cost and availability, and, while they may detect abnormalities associated with disease state, they do not provide insight into molecular mechanism. Their cost alone limits the ability of these tests to be used widespread in a clinical diagnosis. Finally, the time involved to acquire these neuroimages for both clinical staff and patients is significant and cannot be overlooked when considering potential premotor PD biomarkers. Thus, it is likely that the structural/functional neuroimaging methods will be largely a research tool in the area of biomarkers in the near future.

#### Genetic markers

Clearly, identification of premotor biomarkers first requires identification of the population at risk. One obvious choice is those with premotor signs, e.g. RBD and constipation, an option which is, as previously discussed, limited by the non-specific nature of these symptoms prior to the onset of motor symptoms. Another possibility is to follow those with genetic mutations that lead to familial PD. Thus, in the following section, we will address the genetic mutations with a reasonable prevalence for the purpose of premotor biomarker studies: *parkin*, glucocerebrosidase (*GBA*), and leucine-rich repeat kinase-2 (*LRRK2*).

While most idiopathic PD arises *de novo* and without any known genetic mutation, the known genetic aberrations which cause familial PD are invaluable in aiding investigators to elucidate the pathways contributing to clinical diagnosis and the study of preclinical PD biomarkers. Mutations in the *parkin* gene, which encodes a ubiquitin E3 ligase, results in enzymatic activity loss and misfolding [102] and is responsible for the majority of juvenile, early-onset PD [103]. *Parkin* gene mutations may serve as potential premotor biomarker as detecting this mutation in the pre-symptomatic stages of the disease will allow for the evaluation of relevant biomarkers in relation to early-onset PD [104].

Another genetic mutation that has been associated with PD is the gene encoding *GBA.* Mutations in *GBA* cause Gaucher disease, which is the most common autosomal recessive lysosomal storage disease [105], and its relationship with parkinsonism has been reviewed extensively elsewhere [106]. PD and PD-like syndromes associated with *GBA* mutations were first described in 1996 [107], and have subsequently been demonstrated to occur at much higher frequencies in PD patients than controls in pan-ethnic populations from Israel [108,109], Italy [110], China [111,112], North Africa [113], Great Britain [114], Europe [115], and the United States [116,117]. In fact, a large 16-center, multi-country analysis, 15% of Ashkenazi Jewish PD patients had one of two of the most common *GBA* mutations compared to 3% of controls [118]. Furthermore, in almost 2000 non-Jewish PD patients, 7% tested positive for *GBA* mutations after full sequencing of the gene and concluded that patients with *GBA* mutations present with an earlier onset of PD symptoms [118]. Most promisingly, these pan-ethnic population studies are just beginning to be supported by mechanistic studies. For example, one group recently demonstrated that in PD patients with *GBA* mutations, an average of 75% of Lewy Bodies examined were positive for *GBA *[119]. Others have shown that *GBA* mutants can raise the levels of α-synuclein *in vitro* and that the amassing of α-synuclein was reversed by activation of autophagy pathways [120]. Moreover, α-synuclein and *GBA’s* interaction takes place optimally under pH conditions like those in the lysosome [121], thereby further linking PD pathology with to *GBA* function. Thus, *GBA* mutations represent an increased risk for PD development and add additional evidence to a genetic component to the disease.

Mutations in another gene, *LRRK2*, are probably among the most studied. In contrast to *parkin* mutations, *LRRK2* mutations result in an autosomal dominant form of PD that closely resembles idiopathic PD. *LRRK2* mutations account for about 1-2% of sporadic PD cases worldwide, but in genetically isolated populations, such as Ashkenazi Jews and North African Arabs, the mutations can account for upwards of 30-40% of sporadic and familial PD cases [122-127]. The most common mutation is G2019S, which produces a constitutively active kinase [128]. Recent evidence points to *LRRK2* involvement in dopaminergic neuron transmission further linking the gene to PD [129]. Additionally, another recent study examined the downstream targets of *LRRK2* and found that α-synuclein is phosphorylated by *LRRK2,* further implicating α-synuclein in both familial and sporadic PD [130]. This led our group to examine a cohort of symptomatic and asymptomatic *LRRK2* mutation carriers. Because of the high conversion rate of *LRRK2* mutation carriers to clinical disease, this patient population presents a unique subset of PD patients in which evaluation of symptoms and biomarkers can occur decades before clinical signs appear. One of our recent studies attempted to correlate dopaminergic function with both DJ-1 and α-synuclein levels in *LRRK2* mutation carrier CSF. The major finding of this study was that no apparent relationship existed between dopaminergic loss and levels of DJ-1 and α-synuclein in patient CSF [131]. Another recent publication resulting from the same patient cohort [131] investigated *LRRK2* mutation carriers for the levels of amyloid β-42 (Aβ_42_) and tau in patient CSF [132]. We found that *LRRK2* mutation carriers showed reduced CSF Aβ_42_ and tau levels that correlated with lower dopaminergic neuron function as determined by PET tracer [132]. These findings elucidate the possible role DJ-1, α-synuclein, Aβ_42_, and tau in premotor PD and provide insight into which direction future premotor PD biomarker studies might take.

**Figure 2:**
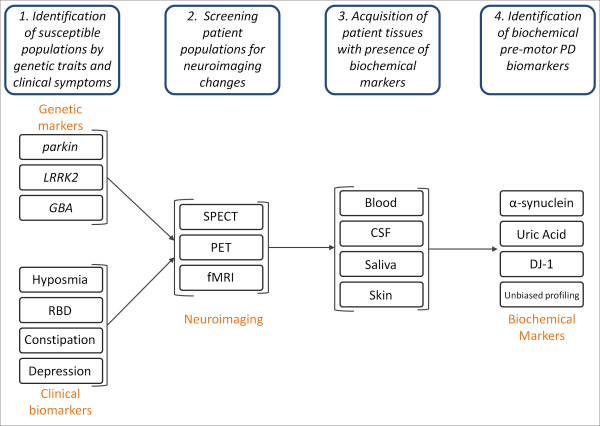
**The Search for Premotor PD Biomarkers.** 1. In order to discover premotor PD biomarkers, it is necessary to first enrich for patient population more likely to develop PD. Patients with genetic susceptibility markers, for example, *parkin* and *LRRK2* mutations, and clinical biomarkers, hyposmia, RBD, constipation, and depression. 2. Patients with genetic markers and clinical phenotypes will undergo screening for neuroimaging changes known to correlate with PD. 3. After verification with neuroimaging, various patient tissues where the presence of biochemical markers has already been verified will be acquired (blood, CSF, saliva, and skin). 4. Premotor PD biomarker assessment and identification will use changes in the levels of known biochemical markers, i.e., α-synuclein, uric acid, DJ-1, in addition to others identified through unbiased profiling.

Other researchers have been able to differentiate between *LRRK2* PD patients and their healthy family members with or without *LRRK2* mutations using metabolomic profiling of *LRRK2*-PD, *LRRK2*-mutation carriers, control, and idiopathic PD patients [133]. Another group was also able to distinguish between asymptomatic carriers of *LRRK2* mutations and healthy carriers by measuring gait alterations [134]. They found that there were significant differences in gait variability measures under three different gait conditions [134] leaving open the possibility that motor symptoms might appear earlier than previously thought. Taken together, these genetic populations that have a high conversion rate from asymptomatic to PD provide a unique cohort of patients to help validate other biomarker discoveries. As these patients can easily be followed in a longitudinal fashion, examining clinical presentation along with biological fluids and assessing them for PD biomarkers will be key in developing our understanding of biomarkers, disease progression, and possible disease interventions (Figure 2). Structural and functional neuroimaging methods will be certainly helpful in determining whether an asymptomatic subject is at premotor stage or not.

In addition to *LRRK2* and *parkin* mutations, the usefulness of having a genetic marker as an *a priori* identifier of at-risk populations has led to attempts to identify other loci associated with PD. Several groups have completed independent genome-wide association studies for PD but have had inconsistent results [135-137]. However, two loci previously associated with neurodegenerative disease, *SNCA* (encoding α-synuclein) and *MAPT* (encoding tau), have been reproducibly implicated in several studies, strongly suggesting a role for both in PD pathogenesis [138-143]. However, PD generation appears to be a complex process requiring the interactions of multiple genetic and environmental factors, so the identification of high-risk groups, prior to the emergence of motor symptoms, remains a significant problem, until a robust premotor biomarker can be found in an easily accessible sample source.

## Conclusions

The need to develop premotor PD biomarkers is increasingly apparent as the aging population grows and considering the limited effectiveness of neuroprotective treatments in altering disease course at the advanced stages. The clinical biomarkers have been well-characterized and correlated with PD outcomes and severity but tend to lack specificity and sensitivity. Neuroimaging, however, can be highly sensitive and specific for premotor PD but significant cost and access barriers exist. The most promising avenue for future work involves both genetic markers and biochemical markers, and currently, efforts are underway at several sites worldwide, including the Michael J. Fox Foundation (http://www.michaeljfox.org/research_MJFFresearchTools.cfm) and the Parkinson’s UK Brain Bank (http://www.parkinsons.org.uk/), to collect and utilize PD tissues to this end [144]. Because some genetically susceptible populations, such as *LRRK2* or *GBA* mutation carriers, possess known PD-associated mutations, they can be employed to validate and screen potential biochemical markers underutilized biological samples, such as saliva. In short, the best outlook for premotor PD biomarkers relies heavily on the rapid and reliable identification of premotor biomarkers with independent validation of biochemical markers the clinical cohort most likely to develop PD.

## Competing interests

The authors declare no competing interests.

## Authors’ contributions

BRH carried out literature searches, reviewed and synthesized current literature, and organized, drafted, and revised the manuscript. THS helped draft and critically revise the manuscript. JZ helped critically revise and gave final approval to the manuscript.
